# Assessment of biofilm resistance, degree of conversion, and mechanical properties of nanomodified 3D-printed orthodontic clear aligner (in-vitro study)

**DOI:** 10.1186/s12903-026-09350-y

**Published:** 2026-07-22

**Authors:** Amal I. Elnaggar, Amal E. Fahmy, Nesrine F. Hanafi, Salma Aboulgheit

**Affiliations:** 1 Dental Biomaterials Department, Faculty of Dentistry, University of Alexandria, Champollion Street – Azarita, Alexandria, 21521 Egypt; 2 Medical Microbiology and Immunology Department, Faculty of Medicine, Alexandria University, Alexandria, Egypt

**Keywords:** Orthodontic clear aligner, 3D printing, Biofilm resistance, Degree of conversion, Flexural strength, Surface microhardness

## Abstract

**Background:**

Three-dimensional (3D)-printed orthodontic clear aligners represent a paradigm shift in malocclusion management; however, they remain susceptible to bacterial biofilm accumulation. Therefore, modification strategies that enhance resistance to biofilm accumulation without compromising mechanical performance are required. This study evaluated biofilm resistance, degree of conversion (DC%), flexural strength (FS), and surface microhardness (VHN) of commercially available 3D-printed clear aligner resin (CR) modified with nanozeolite (NZ) and chitosan nanoparticles (Chs NPs).

**Methods:**

A total of 432 3D-printed specimens were fabricated and allocated into six groups: Group I (control): CR; Group II: (CR + 0.25 wt% NZ); Group III: (CR + 0.5 wt% NZ); Group IV: (CR + 0.25 wt% Chs NPs); Group V: (CR + 0.5 wt% Chs NPs); and Group VI (hybrid): (CR + 0.25 wt% NZ + 0.25 wt% Chs NPs). Surface topography and chemical characterization were performed using scanning electron microscopy (SEM) and Fourier-transform infrared spectroscopy (FTIR) for DC%. Biofilm resistance against *Streptococcus mutans* was assessed using a crystal violet assay by measuring optical density (OD) after 48-hour and 14-day incubation periods. FS and VHN were evaluated using a universal testing machine and a Vickers microhardness tester, respectively. All properties were evaluated before and after 14-day aging. Data were analyzed using two-way ANOVA and independent samples *t*-test with Bonferroni adjustment (*α* = 0.05).

**Results:**

Two-way ANOVA indicated that the interaction between nanoparticle content and aging significantly influenced the DC%, OD, FS, and VHN values (*p* < 0.001). The 0.25 wt% NZ group showed the lowest OD after 48 h, whereas the 0.5 wt% NZ group showed the lowest OD after 14 days. The hybrid group demonstrated the highest FS and VHN values before and after aging. DC% values remained comparable among most groups, except for the 0.5 wt% Chs NPs group, which exhibited significantly lower DC% values before aging. Overall, the 0.5 wt% NZ group demonstrated the most favorable balance between reduced biofilm biomass and mechanical performance.

**Conclusions:**

Nanoparticle incorporation improves the functional properties of 3D-printed clear aligners. NZ-containing groups reduced *Streptococcus mutans* biofilm biomass while preserving acceptable mechanical performance, suggesting potential for development of biofilm-resistant aligner materials.

## Background

 Clear aligner therapy has become widely used in orthodontics as an aesthetic alternative to conventional fixed appliances for the correction of malocclusion. This treatment involves wearing a sequence of transparent plastic trays that fit closely over the teeth and are worn continuously, except during eating and brushing. Each aligner is typically replaced every one to two weeks to achieve the planned tooth movement. The main advantages of clear aligners include their transparency, ease of use, and improved oral hygiene maintenance, allowing patients to undergo orthodontic treatment discreetly while enhancing appearance and self-confidence during treatment [1, 2]. Traditionally, clear aligners have been fabricated using thermoforming techniques. However, this process is time-consuming, technique-sensitive, and associated with unpredictable alterations in aligner properties, in addition to negative environmental impacts [2–4]. Recently, three-dimensional (3D) printing has emerged as an alternative manufacturing approach for clear aligners, enabling in-house fabrication with improved precision, reproducibility, and customizable thickness [2, 5]. Despite these advantages, several limitations remain, including restricted availability of approved materials and software, cytotoxicity, leaching of uncured resin components, colour instability, and increased surface roughness, which may enhance bacterial adhesion and compromise oral hygiene [2, 5–7]. Moreover, prolonged wear of clear aligners may reduce salivary flow and promote biofilm formation within the oral cavity, potentially increasing the risk of periodontal inflammation, incipient caries, and white spot lesions [6, 8, 9]. Therefore, the development of biofilm-resistant clear aligners through coating or incorporation of bioactive nanoparticles has gained increasing research interest [8, 10–12].

Chitosan nanoparticles (Chs NPs) are natural biopolymers derived from chitin, which is primarily obtained from marine shellfish. They are biocompatible, biodegradable, nontoxic, and possess notable antibiofilm and bioactive properties, making them widely applicable in biomedical research [13]. Chs NPs may contribute to reduced bacterial adhesion and biofilm accumulation through electrostatic interaction between positively charged chitosan molecules and anionic sites of bacterial cell membranes, leading to membrane disruption and bacterial cell death [14]. Previous studies have demonstrated that incorporating Chs NPs into dental materials enhances their antimicrobial properties and reduces biofilm formation [15–17]. Furthermore, a previous study reported that incorporating specific concentrations of Chs NPs into 3D-printed aligners improved their antibiofilm activity against *Streptococcus mutans* (*S. mutans*), a primary cariogenic bacterium, without adversely affecting certain physical and mechanical properties [7].

Nanoclay, an aluminosilicate-based material, is among the oldest naturally occurring materials and has been extensively utilized in various industrial and biomedical applications [18, 19]. Previous studies have demonstrated that nanoclay reinforcement improves the mechanical performance of polymeric materials, including polymethyl methacrylate (PMMA)-epoxy composites [20]. In addition, nanoclay incorporation into dental materials has shown promising antimicrobial effects [21, 22]. Nanozeolite (NZ), a biocompatible crystalline porous aluminosilicate composed of tetrahedral aluminate (AlO4) and silicate (SiO4) structures, is a major constituent of clay deposits and is characterized by ion-exchange, adsorption, and catalytic properties [18, 23]. Its antimicrobial efficacy may be enhanced through loading with metal ions possessing potent antimicrobial activity [18, 23]. Previous studies have also demonstrated that NZ incorporation improves the performance of various dental polymers [24]. However, the effects of modifying 3D-printed clear aligners with NZ and Chs NPs have not yet been fully investigated. Therefore, this study aimed to evaluate the effect of incorporating different concentrations of nanozeolite and chitosan nanoparticles into commercially available 3D-printed clear aligner resin on the degree of conversion, flexural strength, and surface microhardness before and after aging in artificial saliva for 14 days at 37 °C. In addition, resistance to *S. mutans* biofilm accumulation was assessed after 48 h and 14 days of incubation.

The null hypothesis was that incorporation of nanozeolite and chitosan nanoparticles into 3D-printed orthodontic clear aligner resin would not produce statistically significant differences in degree of conversion, resistance to *S. mutans* biofilm accumulation, flexural strength, or surface microhardness.

## Methods

### Sample size

Sample size was estimated based on a pilot study and previously published data [25–27], resulting in a total of 432 specimens across all studied parameters. The sample size calculation was based on a study power of 90% and a significance level of 5%. Optical density (OD), representing *S. mutans* biofilm biomass quantified using a crystal violet assay, was selected as the primary outcome variable for sample size estimation. For each outcome measure (OD, FS, and VHN), 144 specimens were fabricated (*n* = 24/group). To account for the destructive nature of the tests, the specimens were equally divided into two subgroups (*n* = 12 each) and evaluated at two distinct time points: biofilm assessment after 48 h and 14 days of *S. mutans* incubation, and FS and VHN evaluation before and after the 14-day aging protocol. Sample size calculation was performed using NCSS 2004 and PASS 2000 software [28].

### Designing and fabrication of specimens

Two types of nanoparticles were incorporated into a 3D-printed clear resin (CR; Dental LT Clear Resin V2, Formlabs, Millbury, OH, USA) at specific weight percentages. Nanozeolite (NZ; average size 45 ± 40 nm; NanoTech Egypt for Photo-Electronics., Egypt) and chitosan nanoparticles (Chs NPs; average size 25 ± 5 nm; Nano Gate Co., Egypt) were blended with the CR in glass beakers to prepare the following six nanocomposite formulations: Group I (control): CR; Group II: (CR + 0.25 wt% NZ); Group III: (CR + 0.5 wt% NZ); Group IV: (CR + 0.25 wt% Chs NPs); Group V: (CR + 0.5 wt % Chs NPs) and Group VI (hybrid): (CR + 0.25 wt% NZ + 0.25 wt% Chs NPs). Each mixture was sonicated for 10 min (T-14; L & R manufacturer, USA), followed by continuous magnetic stirring for 1 h (F91T; Falc, Italy) at room temperature. All mixing procedures were performed under light-restricted conditions to prevent premature polymerization.

Digital specimen design was performed using CAD software (Meshmixer 3.5, Autodesk Inc., USA). Two disc-shaped models were created: 10 mm diameter × 2 mm thickness for surface topography analysis and Vickers microhardness (VHN) evaluation, and 5 mm diameter × 1 mm thickness for biofilm resistance evaluation (OD). Additionally, bar-shaped models (10 mm width × 4 mm thickness × 80 mm length) were designed according to ISO 178:2019 standards for flexural strength (FS) evaluation. After digital design, Standard Tessellation Language (STL) files of the different specimens’ designs were printed using a 3D printer (Form 3B, Formlabs, Somerville, MA, USA) at a layer thickness of 100 μm. Specimens were positioned horizontally on the build platform (0° orientation) to ensure consistent layer distribution. To achieve the required sample size, a total of 144 specimens were fabricated for each test (*n* = 24 per group). After printing, the specimens were rinsed for 20 min in isopropyl alcohol (IPA) (El-Gomhoria Co. for chemicals, Egypt) to ensure removal of residual monomers. This was followed by a 60-minute post-curing cycle at 60 °C (Form 3B, Formlabs, Somerville, MA, USA). After support removal, specimens were refined using wet sandpapers (800–2000 grit) to ensure a standardized finish. Ultimately, all printing and post-processing protocols adhered to the manufacturers’ recommendations [29].

Specimens were then divided into two evaluation periods: biofilm resistance specimens were evaluated after 48 h and 14 days of *S. mutans* incubation. For FS and VHN, testing was conducted before and after a 14-day aging protocol in artificial saliva at 37 °C, simulating two weeks of intraoral aligner use.

### Aging protocol

To simulate the oral environment, specimens were placed in 50 mL conical tubes containing the prepared artificial saliva and stored in an incubator (BST 50 20, VEB MLW Dentalfabrik, Germany) at 37 °C for 14 days. To minimize pH fluctuations, the artificial saliva was refreshed daily [30]. After aging, the specimens were rinsed with distilled water and dried before flexural strength and surface microhardness testing. The composition of the artificial saliva (pH 6.7) was prepared according to a previously published protocol [31].

### Surface topography and distribution of nanoparticles

Representative specimens from each group were sputter-coated with gold (JFC-1300; JEOL) to a thickness of approximately 25 nm and examined using scanning electron microscopy (SEM) at magnifications of × 1000 and × 20 000 with an accelerating voltage of 20 kV. SEM analysis was performed to evaluate surface topography and the distribution of nanoparticles within the 3D-printed clear resin [32].

### Chemical characterization and degree of conversion (DC%) analysis

Representative specimens from each group were ground into powder, and 0.5 g of the specimens was mixed with potassium bromide (KBr) and pressed under vacuum to create pellets. These pellets were analysed using Fourier Transform Infrared Spectroscopy (FTIR; Spectrum Two; PerkinElmer, USA) for chemical characterization and degree of conversion (DC%) analysis. FTIR spectra were recorded 3 times at the wavelength range 4000–400 cm^− 1^, resolution 2 cm^− 1,^ and the average spectrum was used for analysis. The degree of conversion was calculated by measuring the ratio of the absorbance aliphatic C═C peak at 1638 cm^− 1^ to the absorbance carbonyl C═O peak at 1720 cm^− 1^ in both uncured and polymerized states, according to the following formula [33, 34]:

$$\mathrm{DC} \% = \left[1 -\left\{ \left( 1638 \mathrm{cm} ^{-1}/1720 \mathrm{cm} ^{-1} \right) _{\mathrm{Polymerized}} / \left(1638 \mathrm{cm} ^{-1} / 1720 \mathrm{cm} ^{-1}\right) _{\mathrm{Unpolymerized}} \right\} \times 100 \right. $$

### Biofilm resistance assessment using crystal violet assay (CV) in 96-well microplates

For biofilm assessment, disc-shaped specimens (5 mm diameter × 1 mm thickness) were fabricated (*n* = 144) and sterilized using 70% ethanol. The Gram-positive bacterium *Streptococcus mutans (S. mutans)* was obtained from the clinical microbiology laboratory, Faculty of Medicine, Alexandria University, and cultured in brain heart infusion medium (BHI; Oxoid) at 37 °C. Specimens were divided into two equal groups. One group was incubated for 48 h and the other for 14 days in 96-well microplates containing a microbial suspension adjusted to 0.5 McFarland standard (1.5 × 10⁸ CFU/mL). After incubation, the bacterial suspension was gently aspirated, and the specimens were washed three times with 200 µL of phosphate-buffered saline (PBS) to remove non-adherent cells. After air-drying at room temperature, biofilms were fixed with 150 µL of absolute methanol for 15 min, followed by drying at 37 °C for 20 min. Biofilm staining was performed by adding 200 µL of 0.1% crystal violet solution to each well for 20 min at ambient temperature. Two washing cycles were performed to remove unbound dye, and after drying, bound CV was dissolved in 200 µL of 33% glacial acetic acid. After homogenization by pipetting, the optical density (OD) was measured at 570 nm using an ELISA reader (Stat fax-2100 Universal Microplate Reader; Awareness Technology Inc., USA) [9, 35]. All experiments were performed in three independent replicates, and the mean values were used for statistical analysis to ensure reproducibility and accuracy.

### Flexural Strength (FS)

According to the ISO 178:2019 standard, bar-shaped specimens (10 mm width × 4 mm thickness × 80 mm length) were fabricated (*n* = 144) to determine flexural strength (FS; MPa) using a three-point bending test on a universal testing machine (5ST, Tinius Olsen, England) according to the following equation:

$$\mathrm{FS} = 3FL/(2bh^{2})$$

where *F* represents the maximum load (N), while *L*,* b*, and *h* denote the span length (64 mm), the specimen’s width (10 mm), and height (4 mm), respectively. A loading pin with a 5-mm radius was applied to bar-shaped specimens until fracture at a crosshead speed of 1 mm/min [36].

### Surface microhardness (VHN)

Surface microhardness was measured using a Vickers microhardness tester (HVS-1000 A; Jinan Hensgrand Instrument Co., Ltd., China) under a load of 50 gf for 30 s at room temperature. Disc-shaped specimens (10 mm diameter × 2 mm thickness; *n* = 144) were used. Five indentations were generated in each specimen, and then the average of the Vickers hardness number (VHN) was obtained [37].

### Statistical analysis

Data normality was assessed using descriptive statistics, Q–Q plots, histograms, and normality tests. Data were approximately normally distributed; therefore, parametric tests were applied. Results were expressed as means and standard deviations (SD). Percentage change was calculated using the following formula: $\left[\left(\text{Value after} -- \text{Value before} \right) / \text{Value before} \right] \times 100$ Comparisons between the study groups were performed using one-way ANOVA, followed by Bonferroni-adjusted post hoc pairwise comparisons. To evaluate the effects of nanoparticle content and aging protocol, as well as their interaction on the study outcomes (DC%, OD, FS, and VHN), a two-way ANOVA was applied. Adjusted means, standard errors (SE), and 95% confidence intervals (CI) were reported. Within-group comparisons across time points were performed using an independent samples *t*-test due to the destructive nature of the testing, which required separate specimens for each time interval. Statistical significance was set at *p* < 0.05. All analyses were performed using IBM SPSS Statistics for Windows, Version 26.0 (IBM Corp., Armonk, NY, USA).   

## Results

### Surface topography and distribution of nanoparticles

**Fig. 1:**
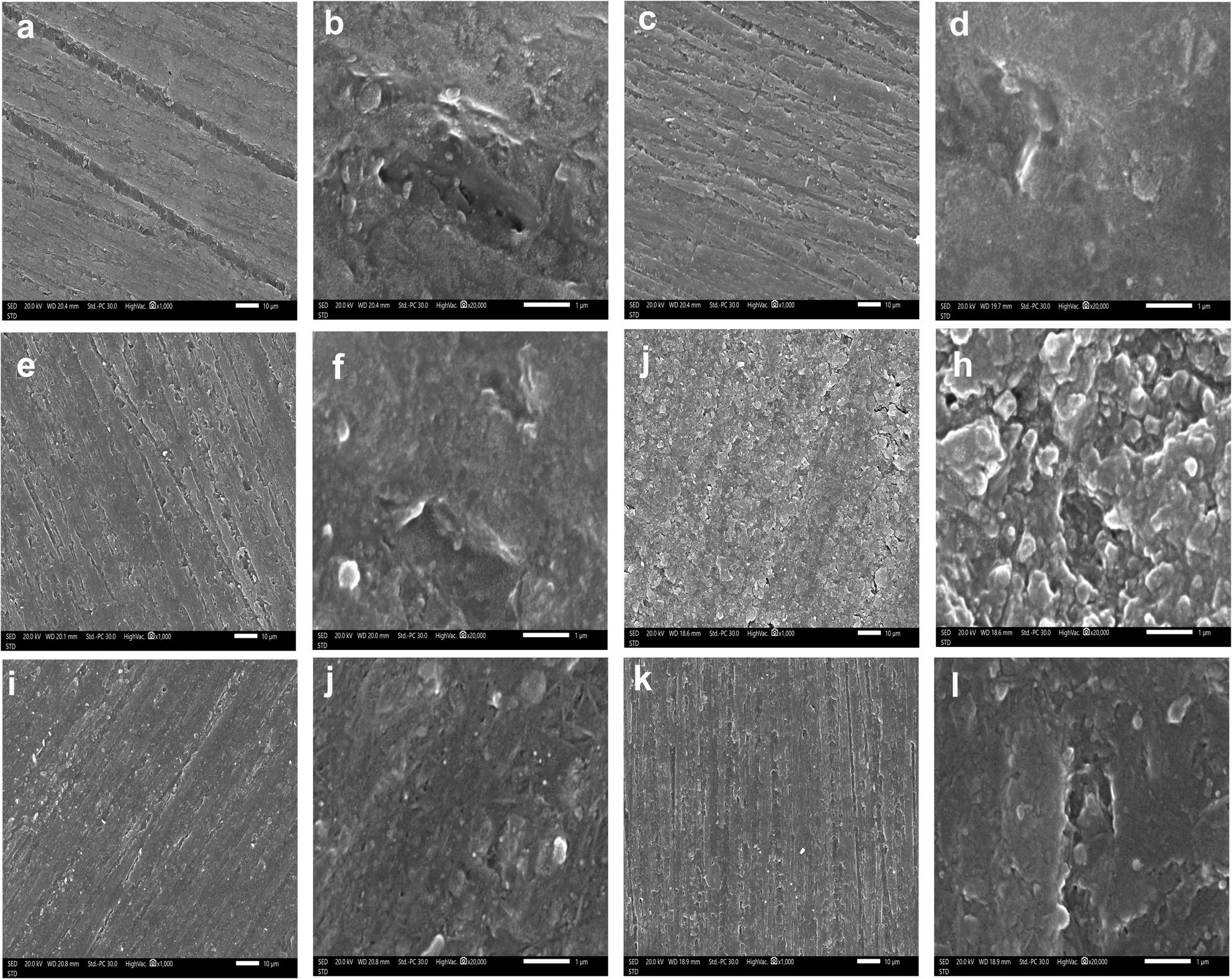
Representative SEM images showing surface topography and nanoparticle distribution within the 3D-printed clear resin. (**a**, **b**) Control group; (**c**, **d**) 0.25 wt% NZ group; (**e**, **f**) 0.5 wt% NZ group; (**g**, **h**) 0.25 wt% Chs NPs group; (**i**, **j**) 0.5 wt% Chs NPs group; and (**k**, **l**) hybrid group. Magnifications: (**a**, **c**, **e**, **g**, **i**, **k**) × 1000 (scale bar = 10 µm) and (**b**, **d**, **f**, **h**, **j**, **l**) × 20 000 (scale bar = 1 µm). NZ: nanozeolite; Chs NPs: chitosan nanoparticles; Hybrid: 0.25 wt% NZ + 0.25 wt% Chs NPs

Representative SEM images (Fig. 1) demonstrated differences in surface topography and nanoparticle distribution among the study groups. The control group exhibited a homogeneous resin matrix with wider porous areas between the printed layers (Fig. 1 a, b). Groups containing NZ demonstrated relatively smooth and uniform surfaces with fewer porosities and minimal nanoparticle aggregation (Fig. 1 c–f). Nanoparticles were primarily distributed between the printed layers. The 0.25 wt% Chs NPs group exhibited distinct clustered bright areas, suggesting enhanced polymer crosslinking within the resin matrix (Fig. 1 g, h). In contrast, the 0.5 wt% Chs NPs group (Fig. 1 i, j) and the hybrid group (Fig. 1 k, l) showed increased nanoparticle aggregation.

### Chemical characterization and degree of conversion (DC%) analysis

**Fig. 2:**
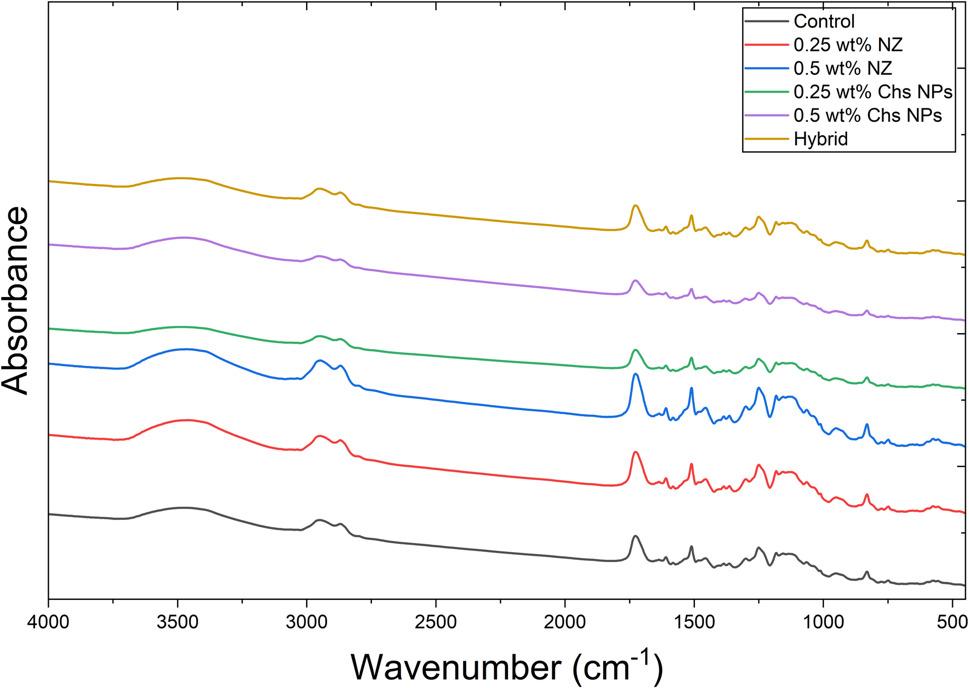
Fourier Transform Infrared Spectroscopy (FTIR) spectra of the control (CR), 0.25 wt% NZ, 0.5 wt% NZ, 0.25 wt% Chs NPs, 0.5 wt% Chs NPs, and hybrid groups. NZ: nanozeolite; Chs NPs: chitosan nanoparticles; Hybrid: 0.25 wt% NZ + 0.25 wt% Chs NPs

Representative FTIR spectra of all groups (Fig. 2) demonstrated the characteristic absorption bands of the resin matrix, including O–H/N–H (3445 cm⁻¹), C–H (2953 and 2866 cm⁻¹), C = O (1727 cm⁻¹), C = C (1637 cm⁻¹), C–N–H (1511 cm⁻¹), and C–O (1250, 1110 cm⁻¹), corresponding to Bisphenol A dimethacrylate, methacrylate, and urethane dimethacrylate components [26]. No additional peaks related to nanoparticle incorporation were detected, likely due to the relatively low nanoparticle concentrations. However, broadening of the O–H/N–H absorption band was observed in the NZ-containing groups, with a shift toward higher wavenumbers in the 0.5 wt% NZ, 0.25 wt% Chs NPs, 0.5 wt% Chs NPs, and hybrid groups. These spectral changes may indicate enhanced hydrogen bonding and physical interactions between the nanoparticles and resin matrix rather than chemical modification.

#### Degree of conversion (DC%)

**Fig. 3:**
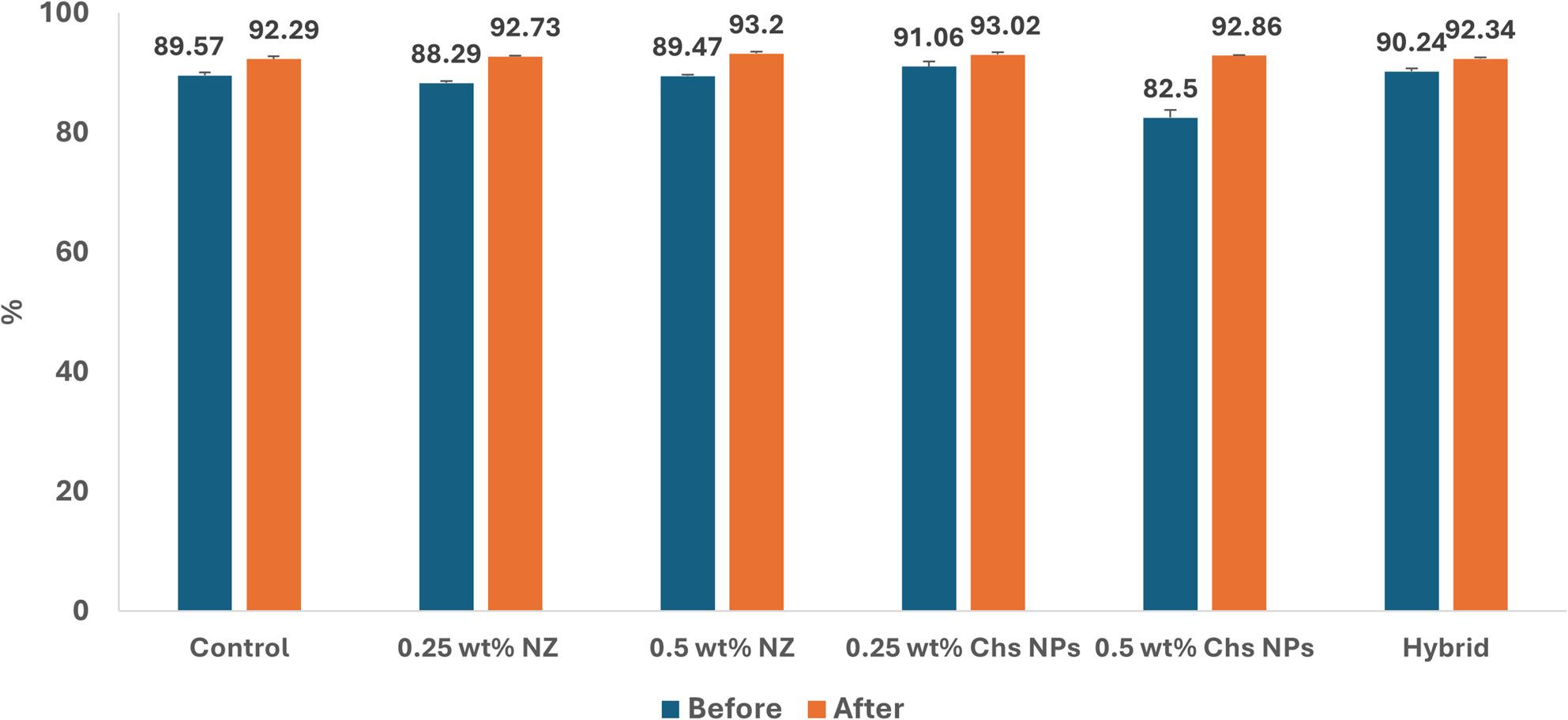
Comparison of degree of conversion (DC%) among the study groups before and after the aging protocol. NZ: Nanozeolite; Chs NPs: Chitosan nanoparticles; Hybrid: 0.25 wt% Chs NPs + 0.25 wt% NZ

Two-way ANOVA results (Table 3) demonstrated a statistically significant interaction between nanoparticle content and the aging protocol on DC% values (*p* < 0.001). As presented in Tables 1 and 2; Fig. 3, most modified groups exhibited DC% values comparable to those of the control group. However, the 0.5 wt% Chs NPs group demonstrated the lowest DC% before aging (mean ± SD: 82.50 ± 1.28) and showed the highest percentage increase after aging (12.57 ± 1.61%). The 0.25 wt% Chs NPs group exhibited the highest DC% before aging (mean ± SD: 91.06 ± 0.81), whereas the 0.5 wt% NZ group demonstrated the highest DC% after aging (mean ± SD: 93.20 ± 0.32).

**Table 1:** Comparison of degree of conversion (DC%) among the study groups before and after the aging protocol

	Control	0.25 wt% NZ	0.5 wt% NZ	0.25 wt% Chs NPs	0.5 wt% Chs NPs	0.25 wt% Chs NPs + 0.25 wt% NZ	*p* value 1
Mean ± SD							
Before	89.57 ± 0.52 ^a^	88.29 ± 0.34 ^a^	89.47 ± 0.19 ^a^	91.06 ± 0.81 ^a^	82.50 ± 1.28 ^b^	90.24 ± 0.48 ^a^	**< 0.001** ^*^
After	92.29 ± 0.45 ^a, b^	92.73 ± 0.16 ^a, b^	93.20 ± 0.32 ^a, b^	93.02 ± 0.42 ^a^	92.86 ± 0.13 ^a, b^	92.34 ± 0.19 ^b^	**0.02** ^*^
% change	3.04 ± 1.06 ^a^	5.03 ± 0.31 ^a^	4.17 ± 0.56 ^a^	2.15 ± 1.12 ^a^	12.57 ± 1.61 ^b^	2.33 ± 0.58 ^a^	**< 0.001** ^*^
***p*** **value 2**	**0.04** ^*^	**0.001** ^*^	**0.006** ^*^	**0.04** ^*^	**0.004** ^*^	**0.02** ^*^	

*NZ* Nanozeolite, *Chs NPs* Chitosan nanoparticles

*SD* standard deviation

*p* value 1: Comparison among the study groups using one-way ANOVA

*p* value 2: Comparison between values before and after the aging protocol within each group using an independent samples t-test.

*Statistically significant at *p* value < 0.05

a-e: different letters denote significant differences among groups according to Bonferroni-adjusted pairwise comparisons.

**Table 2:** Post-hoc pairwise comparisons of degree of conversion (DC%) among the study groups before and after the aging protocol

Group	Compared to	Before	After	Difference	
		*p* value			
Control	0.25 wt% NZ	0.70	1.00	0.43	
	0.5 wt% NZ	1.00	0.10	1.00	
	0.25 wt% Chs NPs	0.34	0.19	1.00	
	0.5 wt% Chs NPs	**< 0.001** ^*^	0.61	**< 0.001** ^*^	
	0.25 wt% Chs NPs + 0.25 wt% NZ	1.00	1.00	1.00	
0.25 wt% NZ	0.5 wt% NZ	0.93	1.00	1.00	
	0.25 wt% Chs NPs	0.10	1.00	0.10	
	0.5 wt% Chs NPs	**< 0.001** ^*^	1.00	**< 0.001** ^*^	
	0.25 wt% Chs NPs + 0.25 wt% NZ	0.10	1.00	0.10	
0.5 wt% NZ	0.25 wt% Chs NPs	0.25	1.00	0.40	
	0.5 wt% Chs NPs	**< 0.001** ^*^	1.00	**< 0.001** ^*^	
	0.25 wt% Chs NPs + 0.25 wt% NZ	1.00	0.04*	0.59	
0.25 wt% Chs NPs	0.5 wt% Chs NPs	**< 0.001** ^*^	1.00	**< 0.001** ^*^	
	0.25 wt% Chs NPs + 0.25 wt% NZ	1.00	0.28	1.00	
0.5 wt% Chs NPs	0.25 wt% Chs NPs + 0.25 wt% NZ	**< 0.001** ^*^	0.85	**< 0.001** ^*^	

*NZ* Nanozeolite, *Chs NPs* Chitosan nanoparticles

*Statistically significant according to Bonferroni-adjusted post hoc comparisons (*p* < 0.05)

**Table 3:** Two-way ANOVA evaluating the effects of nanoparticle content and aging protocol on degree of conversion (DC%)

		Adjusted mean (SE)	95% CI	*p* value
Nanoparticle content	Control	90.93 (0.21) ^**a, b**^	90.47, 91.39	**< 0.001** ^*^
	0.25 wt% NZ	90.51 (0.21) ^a^	90.05, 90.97	
	0.5 wt% NZ	91.34 (0.21) ^b^	90.88, 91.80	
	0.25 wt% Chs NPs	92.04 (0.21) ^c^	91.58, 92.50	
	0.5 wt% Chs NPs	87.68 (0.21) ^d^	87.22, 88.15	
	0.25 wt% Chs NPs + 0.25 wt% NZ	91.29 (0.21) ^b^	90.82, 91.75	
Aging protocol	Before	88.52 (0.17)	88.16, 88.88	**< 0.001** ^*^
	After	92.74 (0.07)	92.58, 92.90	

*NZ* Nanozeolite, *Chs NPs* Chitosan nanoparticles

*Statistically significant according to Bonferroni-adjusted post hoc comparisons (*p* < 0.05)

### Optical density of *S. mutans* biofilm biomass (OD)

**Fig. 4:**
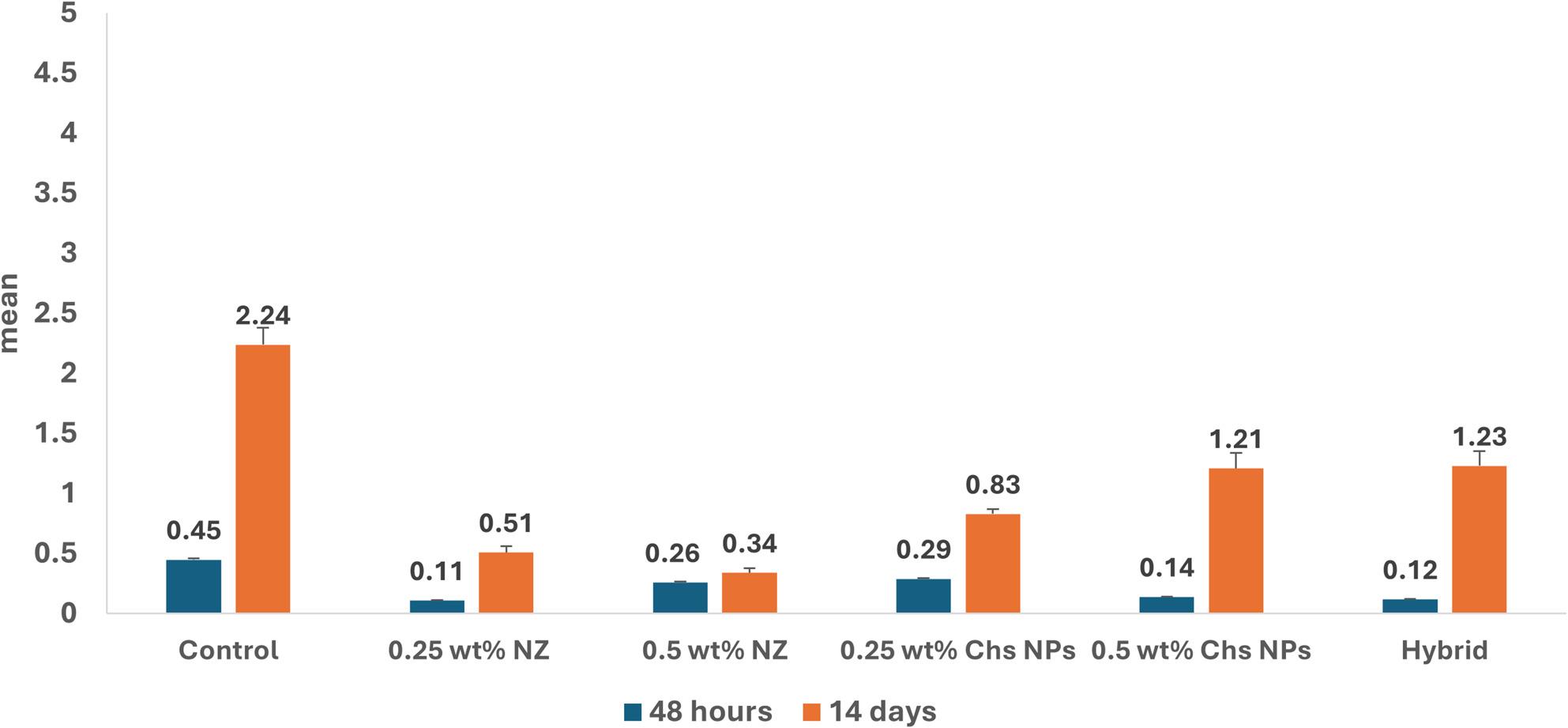
Comparison of optical density (OD) among the study groups at different time intervals. NZ: Nanozeolite; Chs NPs: Chitosan nanoparticles; Hybrid: 0.25 wt% Chs NPs + 0.25 wt% NZ

Regarding OD, two-way ANOVA results (Table 9) demonstrated a statistically significant interaction between nanoparticle content and the aging protocol (*p* < 0.001). As illustrated in Tables 4 and 5; Fig. 4, the control group exhibited the highest OD values following *S. mutans* incubation after both 48 h (mean ± SD: 0.45 ± 0.01) and 14 days (mean ± SD: 2.24 ± 0.14). In contrast, the 0.25 wt% NZ group demonstrated the lowest OD after 48 h (mean ± SD: 0.11 ± 0.002), whereas the 0.5 wt% NZ group exhibited the lowest OD after 14 days of incubation (mean ± SD: 0.34 ± 0.04), with the lowest percentage increase (28.78 ± 16.94%) over the incubation period.

**Table 4:** Comparisons of optical density among the study groups at different time intervals

	Control	0.25 wt% NZ	0.5 wt% NZ	0.25 wt% Chs NPs	0.5 wt% Chs NPs	0.25 wt% Chs NPs + 0.25wt% NZ	*p* value 1
	Mean ± SD						
48 h	0.45 ± 0.01 ^a^	0.11 ± 0.002 ^b^	0.26 ± 0.005 ^c^	0.29 ± 0.008 ^d^	0.14 ± 0.002 ^b^	0.12 ± 0.003 ^b^	**< 0.001** ^*^
14 days	2.24 ± 0.14 ^a^	0.51 ± 0.05 ^b^	0.34 ± 0.04 ^c^	0.83 ± 0.04 ^d^	1.21 ± 0.13 ^e^	1.23 ± 0.12 ^e^	**< 0.001** ^*^
% change	403.43 ± 37.64 ^a^	344.27 ± 43.34 ^a^	28.78 ± 16.94 ^b^	190.36 ± 16.63 ^c^	792.54 ± 101.42 ^d^	907.90 ± 110.08 ^e^	**< 0.001** ^*^
***p*** **value 2**	**< 0.001** ^*^	**< 0.001** ^*^	**< 0.001** ^*^	**< 0.001** ^*^	**< 0.001** ^*^	**< 0.001** ^*^	

*NZ* Nanozeolite, *Chs NPs* Chitosan nanoparticles, *SD* standard deviation

*p* value 1: Comparisons between the study groups using one-way ANOVA

*p* value 2: Comparisons between values obtained after 48 hours and 14 days of S. mutans incubation within each group using an independent samples t-test

*Statistically significant at *p* value < 0.05

a-e: different letters denote significant differences between groups according to Bonferroni-adjusted post hoc comparisons

**Table 5:** Post-hoc pairwise comparisons of optical density (OD) among the study groups at different time intervals

Group	Compared to	48 h	14 days	Difference	
		*p* value			
Control	0.25 wt% NZ	**< 0.001** ^*^	**< 0.001** ^*^	0.77	
	0.5 wt% NZ	**< 0.001** ^*^	**< 0.001** ^*^	**< 0.001** ^*^	
	0.25 wt% Chs NPs	**< 0.001** ^*^	**< 0.001** ^*^	**< 0.001** ^*^	
	0.5 wt% Chs NPs	**< 0.001** ^*^	**< 0.001** ^*^	**< 0.001** ^*^	
	0.25 wt% Chs NP + 0.25 wt% NZ	**< 0.001** ^*^	**< 0.001** ^*^	**< 0.001** ^*^	
0.25 wt% NZ	0.5 wt% NZ	**< 0.001** ^*^	**0.001** ^*^	**< 0.001** ^*^	
	0.25 wt% Chs NPs	**< 0.001** ^*^	**< 0.001** ^*^	**< 0.001** ^*^	
	0.5 wt% Chs NPs	0.10	**< 0.001** ^*^	**< 0.001** ^*^	
	0.25 wt% Chs NPs + 0.25 wt% NZ	1.00	**< 0.001** ^*^	**< 0.001** ^*^	
0.5 wt% NZ	0.25 wt% Chs NPs	**< 0.001** ^*^	**< 0.001** ^*^	**< 0.001** ^*^	
	0.5 wt% Chs NPs	**< 0.001** ^*^	**< 0.001** ^*^	**< 0.001** ^*^	
	0.25 wt% Chs NPs + 0.25 wt% NZ	**< 0.001** ^*^	**< 0.001** ^*^	**< 0.001** ^*^	
0.25 wt% Chs NPs	0.5 wt% Chs NPs	**< 0.001** ^*^	**< 0.001** ^*^	**< 0.001** ^*^	
	0.25 wt% Chs NPs + 0.25 wt% NZ	**< 0.001** ^*^	**< 0.001** ^*^	**< 0.001** ^*^	
0.5 wt% Chs NPs	0.25 wt% Chs NPs + 0.25 wt% NZ	0.39	1.00	**0.001** ^*^	

*NZ* Nanozeolite, *Chs NPs* Chitosan nanoparticles

*Statistically significant according to Bonferroni-adjusted post hoc comparisons (*p* < 0.05)

**Table 6:** Two-way ANOVA evaluating the effects of nanoparticle content and aging protocol on optical density

		Adjusted mean (SE)	95% CI	*p* value
Nanoparticle content	Control	1.34 (0.01) ^a^	1.32, 1.37	**<0.001** ^*^
	0.25 wt% NZ	0.31 (0.01) ^b^	0.28, 0.34	
	0.5 wt% NZ	0.30 (0.01) ^b^	0.27, 0.33	
	0.25 wt% Chs NPs	0.56 (0.01) ^c^	0.53, 0.59	
	0.5 wt% Chs NPs	0.67 (0.01) ^d^	0.65, 0.70	
	0.25 wt% Chs NPs + 0.25 wt% NZ	0.68 (0.01) ^d^	0.65, 0.70	
Aging protocol	48 hours	0.28 (0.002)	0.27, 0.28	**<0.001** ^*^
	14 days	0.92 (0.01)	0.89, 0.94	

*NZ* Nanozeolite, *Chs NPs* Chitosan nanoparticles, *SE* Standard Error, *CI* Confidence Interval

*p* value for the interaction between nanoparticle content and aging protocol: < 0.001*

a-d: different letters denote significant differences among groups according to Bonferroni-adjusted post hoc comparisons

### Flexural strength (FS)

**Fig. 5:**
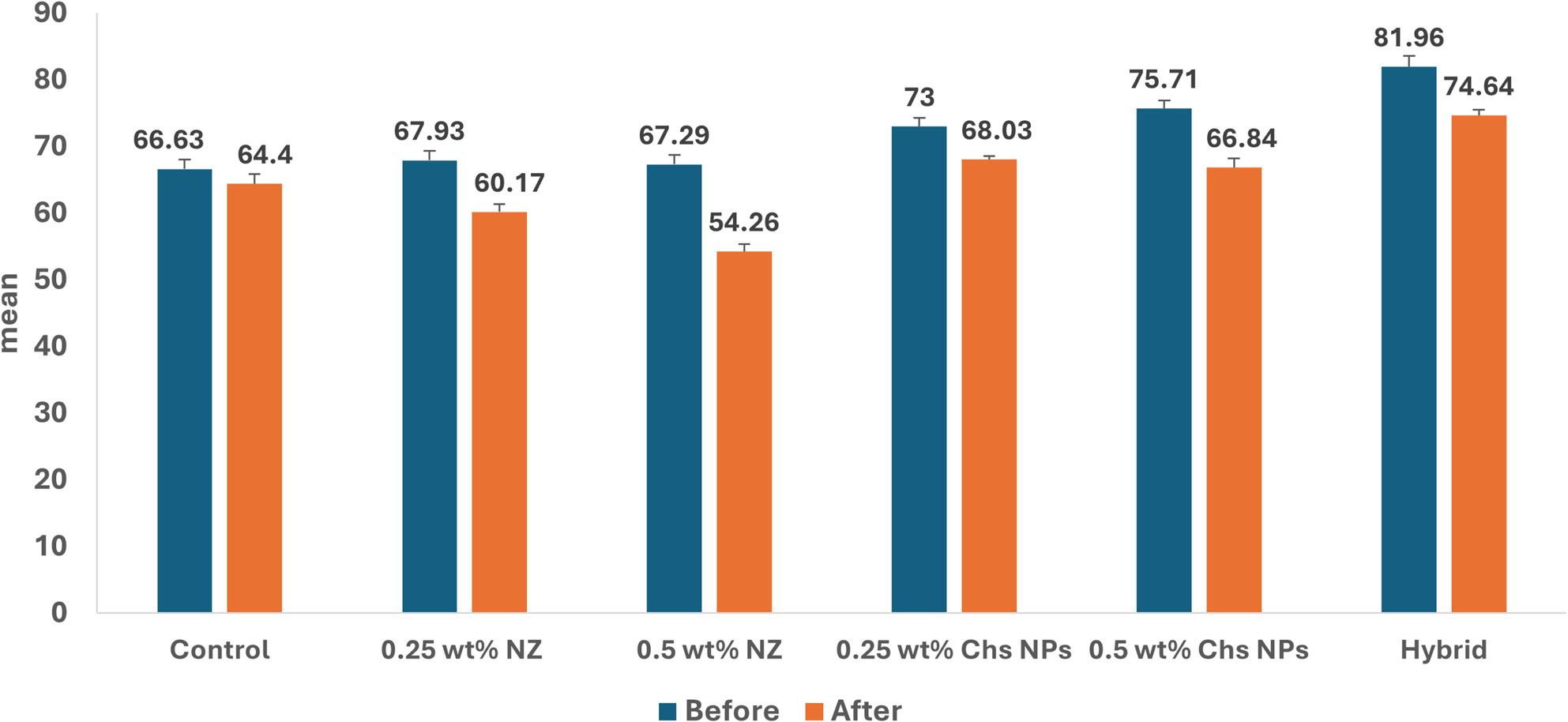
Comparison of flexural strength (MPa) among the study groups before and after the aging protocol. NZ: Nanozeolite; Chs NPs: Chitosan nanoparticles; Hybrid: 0.25 wt% Chs NPs + 0.25 wt% NZ

Regarding FS, Two-way ANOVA results (Table 9) indicated a significant statistical interaction between the aging protocol and nanoparticle content (*p* < 0.001). As illustrated in Tables 7 and 8; Fig. 5, the hybrid group(0.25 wt% Chs NPs + 0.25 wt% NZ) exhibited the highest FS both before(mean ± SD: 81.96 ± 1.62 MPa) and after aging (mean ± SD: 74.64 ± 0.88 MPa). NZ-containing groups showed FS values comparable to the control group before aging. However, the 0.5 wt% NZ group exhibited the highest percentage decrease (-19.33 ± 2.64%) after the aging protocol. In contrast, the control group demonstrated the most stable performance, with the lowest percentage decrease (-3.30 ± 3.37%) after aging.

**Table 7:** Comparisons of flexural strength (MPa) among the study groups before and after the aging protocol

	Control	0.25 wt% NZ	0.5 wt% NZ	0.25 wt% Chs NPs	0.5 wt% Chs NPs	0.25 wt% Chs NPs + 0.25 wt% NZ	*p* value 1
	Mean ± SD						
Before	66.63 ± 1.37 ^a^	67.93 ± 1.38 ^a^	67.29 ± 1.39 ^a^	73.00 ± 1.24 ^b^	75.71 ± 1.17 ^c^	81.96 ± 1.62 ^d^	**< 0.001** ^*^
After	64.40 ± 1.48 ^a^	60.17 ± 1.16 ^b^	54.26 ± 1.12 ^c^	68.03 ± 0.48 ^d^	66.84 ± 1.32 ^d^	74.64 ± 0.88 ^e^	**< 0.001** ^*^
% change	-3.30 ± 3.37 ^a^	-11.40 ± 1.73 ^b^	-19.33 ± 2.64 ^c^	-6.78 ± 1.59 ^d^	-11.70 ± 2.20 ^b^	-8.91 ± 1.54 ^b, d^	**< 0.001** ^*^
***p*** **value 2**	**0.007** ^*^	**< 0.001** ^*^	**< 0.001** ^*^	**< 0.001** ^*^	**< 0.001** ^*^	**< 0.001** ^*^	

*NZ* Nanozeolite, *Chs NPs* Chitosan nanoparticles, *SD* standard deviation

*p* value 1: Comparison among the study groups using one-way ANOVA

*p* value 2: Comparison between values before and after the aging protocol within each group using an independent samples t-test

*Statistically significant at *p* value < 0.05

a-e: different letters denote significant differences among groups according to Bonferroni-adjusted pairwise comparisons

**Table 8:** Post-hoc pairwise comparisons of flexural strength among the study groups before and after the aging protocol

Group	Compared to	Before	After	Difference	
		*p* value			
Control	0.25 wt% NZ	0.34	**< 0.001** ^*^	**< 0.001** ^*^	
	0.5 wt% NZ	1.00	**< 0.001** ^*^	**< 0.001** ^*^	
	0.25 wt% Chs NPs	**< 0.001** ^*^	**< 0.001** ^*^	**0.006** ^*^	
	0.5 wt% Chs NPs	**< 0.001** ^*^	**< 0.001** ^*^	**< 0.001** ^*^	
	0.25 wt% Chs NPs + 0.25 wt% NZ	**< 0.001** ^*^	**< 0.001** ^*^	**< 0.001** ^*^	
0.25 wt% NZ	0.5 wt% NZ	1.00	**< 0.001** ^*^	**< 0.001** ^*^	
	0.25 wt% Chs NPs	**< 0.001** ^*^	**< 0.001** ^*^	**< 0.001** ^*^	
	0.5 wt% Chs NPs	**< 0.001** ^*^	**< 0.001** ^*^	1.00	
	0.25 wt% Chs NPs + 0.25 wt% NZ	**< 0.001** ^*^	**< 0.001** ^*^	0.14	
0.5 wt% NZ	0.25 wt% Chs NPs	**< 0.001** ^*^	**< 0.001** ^*^	**< 0.001** ^*^	
	0.5 wt% Chs NPs	**< 0.001** ^*^	**< 0.001** ^*^	**< 0.001** ^*^	
	0.25 wt% Chs NPs + 0.25 wt% NZ	**< 0.001** ^*^	**< 0.001** ^*^	**< 0.001** ^*^	
0.25 wt% Chs NPs	0.5 wt% Chs NPs	**< 0.001** ^*^	0.17	**< 0.001** ^*^	
	0.25 wt% Chs NPs + 0.25wt% NZ	**< 0.001** ^*^	**< 0.001** ^*^	0.38	
0.5 wt% Chs NPs	0.25 wt% Chs NPs + 0.25 wt% NZ	**< 0.001** ^*^	**< 0.001** ^*^	0.10	

*NZ* Nanozeolite, *Chs NPs* Chitosan nanoparticles.

*Statistically significant according to Bonferroni-adjusted post hoc comparisons (*p* < 0.05)

**Table 9:** Two-way ANOVA evaluating the effects of nanoparticle content and aging protocol on flexural strength

		Adjusted mean (SE)	95% CI	*p* value
Nanoparticle content	Control	65.51 (0.26) ^a^	64.99, 66.04	**< 0.001** ^*^
	0.25 wt% NZ	64.05 (0.26) ^b^	63.53, 64.58	
	0.5 wt% NZ	60.78 (0.26) ^c^	60.25, 61.30	
	0.25 wt% Chs NPs	70.52 (0.26) ^d^	69.99, 71.04	
	0.5 wt% Chs NPs	71.27 (0.26) ^d^	70.75, 71.80	
	0.25 wt% Chs NPs + 0.25 wt% NZ	78.30 (0.26) ^e^	77.78, 78.83	
Aging protocol	Before	72.09 (0.16)	71.77, 72.41	**< 0.001** ^*^
	After	64.72 (0.13)	64.46, 64.99	

*NZ* Nanozeolite, *Chs NPs,* Chitosan nanoparticles, *SE* Standard Error, *CI* Confidence Interval

*p* value for the interaction between nanoparticle content and aging protocol: < 0.001*

a-d: different letters denote significant differences between groups according to Bonferroni-adjusted post hoc comparisons

### Surface microhardness (VHN)

**Fig. 6:**
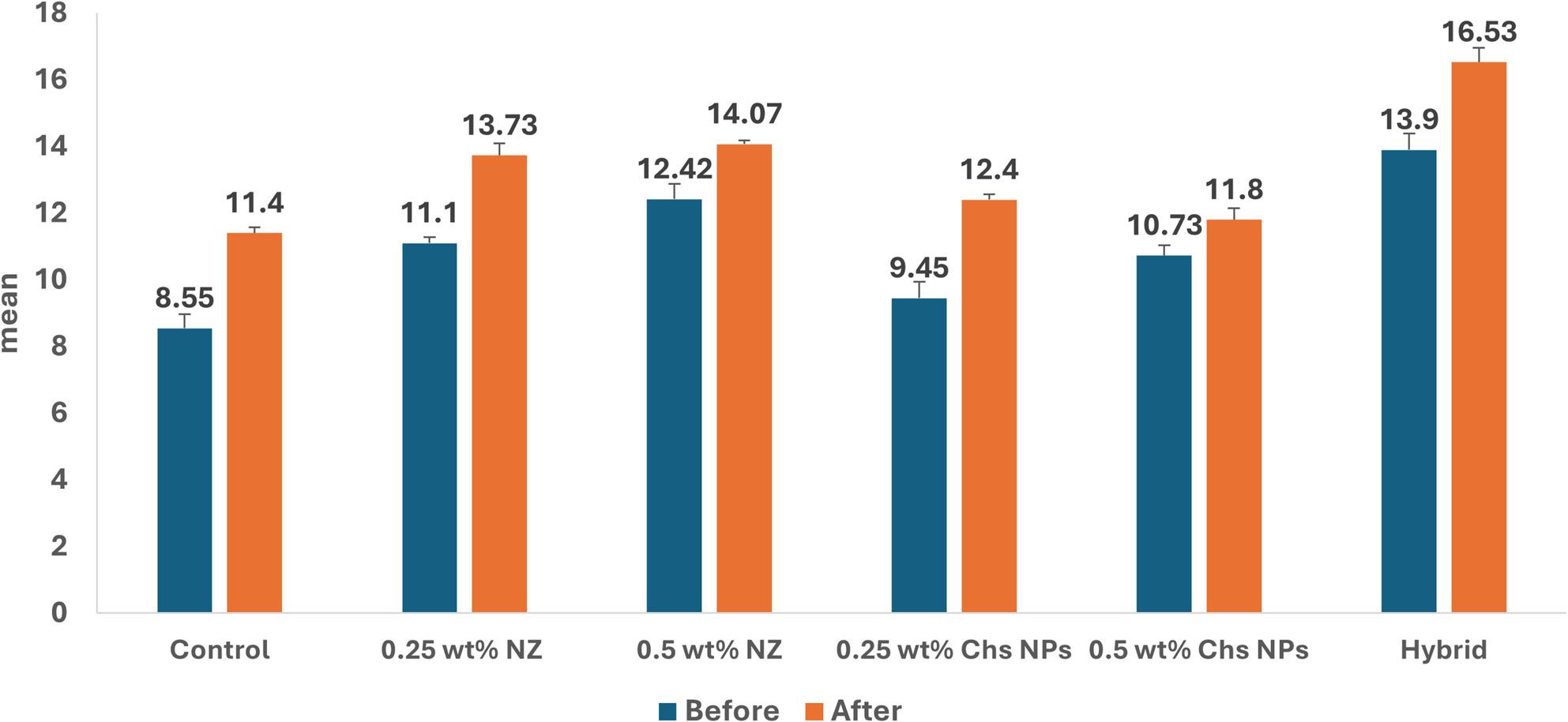
Comparison of surface microhardness among the study groups before and after the aging protocol. NZ: Nanozeolite; Chs NPs: Chitosan nanoparticles; Hybrid: 0.25 wt% Chs NPs + 0.25 wt% NZ

Regarding VHN, Two-way ANOVA results (Table 12) indicated a significant statistical interaction between the aging protocol and nanoparticle content (*p* < 0.001). As illustrated in Tables 10, 11 and 12; Fig. 6, the hybrid group (0.25 wt% Chs NPs + 0.25 wt% NZ) exhibited the highest VHN both before and after aging protocol (mean ± SD: 13.90 ± 0.78 and 16.53 ± 0.43, respectively). In contrast, the control group showed the lowest VHN values before and after aging (mean ± SD: 8.55 ± 0.42 and 11.40 ± 0.17, respectively), while also demonstrating the highest percentage increase (33.62 ± 6.62%) after the aging protocol.

**Table 10:** Comparisons of surface microhardness (VHN) among the study groups before and after the aging protocol

	Control	0.25 wt% NZ	0.5 wt% NZ	0.25 wt% Chs NPs	0.5 wt% Chs NPs	0.25 wt% Chs NPs + 0.25 wt% NZ	*p* value 1
	Mean ± SD						
Before	8.55 ± 0.42 ^a^	11.10 ± 0.40 ^b^	12.42 ± 0.46 ^c^	9.45 ± 0.49 ^d^	10.73 ± 0.31 ^b^	13.90 ± 0.78 ^e^	**< 0.001** ^*^
After	11.40 ± 0.17 ^a^	13.73 ± 0.36 ^b^	14.07 ± 0.10 ^b^	12.40 ± 0.17 ^c^	11.80 ± 0.34 ^d^	16.53 ± 0.43 ^e^	**< 0.001** ^*^
% change	33.62 ± 6.62 ^a^	23.90 ± 6.04 ^b^	13.44 ± 4.46 ^c, e^	31.55 ± 7.20 ^d^	10.02 ± 4.39 ^e^	19.30 ± 7.52 ^b, c^	**< 0.001** ^*^
***p*** **value 2**	**< 0.001** ^*^	**< 0.001** ^*^	**< 0.001** ^*^	**< 0.001** ^*^	**< 0.001** ^*^	**< 0.001** ^*^	

*NZ* Nanozeolite, *Chs NPs* Chitosan nanoparticles, *SD* standard deviation

*p* value 1: Comparison among the study groups using one-way ANOVA

*p* value 2: Comparison between values before and after the aging protocol within each group using an independent samples t-test

*Statistically significant at *p* value < 0.05

a-e: different letters denote significant differences among groups according to Bonferroni-adjusted pairwise comparisons

**Table 11:** Post-hoc pairwise comparisons of surface microhardness (VHN) among the study groups before and after the aging protocol

Group	Compared to	Before	After	Difference	
		*p* value			
Control	0.25 wt% NZ	**< 0.001** ^*^	**< 0.001** ^*^	**0.004** ^*^	
	0.5 wt% NZ	**< 0.001** ^*^	**< 0.001** ^*^	**< 0.001** ^*^	
	0.25 wt% Chs NPs	**0.001** ^*^	**< 0.001** ^*^	**< 0.001** ^*^	
	0.5 wt% Chs NPs	**< 0.001** ^*^	**0.02** ^*^	**< 0.001** ^*^	
	0.25 wt% Chs NPs + 0.25 wt% NZ	**< 0.001** ^*^	**< 0.001** ^*^	**< 0.001** ^*^	
0.25 wt% NZ	0.5 wt% NZ	**< 0.001** ^*^	0.10	**0.001** ^*^	
	0.25 wt% Chs NPs	**< 0.001** ^*^	**< 0.001** ^*^	**0.04** ^*^	
	0.5 wt% Chs NPs	1.00	**< 0.001** ^*^	**< 0.001** ^*^	
	0.25 wt% Chs NPs + 0.25 wt% NZ	**< 0.001** ^*^	**< 0.001** ^*^	1.00	
0.5 wt% NZ	0.25 wt% Chs NPs	**< 0.001** ^*^	**< 0.001** ^*^	**< 0.001** ^*^	
	0.5 wt% Chs NPs	**< 0.001** ^*^	**< 0.001** ^*^	1.00	
	0.25 wt% Chs NPs + 0.25 wt% NZ	**< 0.001** ^*^	**< 0.001** ^*^	0.34	
0.25 wt% Chs NPs	0.5 wt% Chs NPs	**< 0.001** ^*^	**< 0.001** ^*^	**< 0.001** ^*^	
	0.25 wt% Chs NPs + 0.25 wt% NZ	**< 0.001** ^*^	**< 0.001** ^*^	**< 0.001** ^*^	
0.5 wt% Chs NPs	0.25 wt% Chs NP + 0.25 wt% NZ	**< 0.001** ^*^	**< 0.001** ^*^	**0.007** ^*^	

*NZ* Nanozeolite, *Chs NPs* Chitosan nanoparticles

*Statistically significant according to Bonferroni-adjusted post hoc comparisons (*p* < 0.05)

**Table 12:** Two-way ANOVA evaluating the effects of nanoparticle content and aging protocol on surface microhardness (VHN )

		Adjusted mean (SE)	95% CI	*p* value
Nanoparticle content	Control	9.90 (0.08) ^a^	9.81, 10.14	**< 0.001** ^*^
	0.25 wt% NZ	12.42 (0.08) ^b^	12.25, 12.58	
	0.5 wt% NZ	13.24 (0.08) ^c^	13.08, 13.40	
	0.25 wt% Chs NPs	10.93 (0.08) ^d^	10.76, 11.09	
	0.5 wt% Chs NPs	11.27 (0.08) ^d^	11.10, 11.43	
	0.25 wt% Chs NPs + 0.25 wt% NZ	15.22 (0.08) ^e^	15.05, 15.38	
Aging protocol	Before	11.03 (0.06)	10.91, 11.14	**< 0.001** ^*^
	After	13.32 (0.03)	13.26, 13.39	

*NZ* Nanozeolite, *Chs NPs* Chitosan nanoparticles, *SE* Standard Error, *CI* Confidence Interval

*p* value for the interaction between nanoparticle content and aging protocol: < 0.001*

a-d: different letters denote significant differences between groups according to Bonferroni-adjusted post hoc comparisons.

## Discussion

The null hypothesis, stating that incorporation of nanozeolite and chitosan nanoparticles into 3D-printed orthodontic clear aligner resin would not produce statistically significant differences in degree of conversion, resistance to *S. mutans* biofilm accumulation, flexural strength, or surface microhardness, was rejected.

In the present study, nanoparticle concentrations were limited to 0.25 wt% and 0.5 wt% to achieve a balance between *S. mutans* biofilm resistance and preservation of mechanical integrity and optical stability. This conservative selection was guided by previous literature [38], which indicates that higher nanoparticle loadings may promote particle aggregation. Such agglomerates can act as stress concentration sites, potentially facilitating crack propagation and compromising the long-term structural performance of the aligners. In addition, excessive nanoparticle loading may increase light scattering within the resin matrix, which could negatively affect transparency, a critical aesthetic property in clear aligner therapy [2]. However, optical performance was not quantitatively evaluated in this study and should be addressed in future investigations to confirm clinical relevance.

Degree of conversion (DC%) is a critical parameter influencing the biocompatibility, mechanical behaviour, and physical properties of resin-based materials, as it reflects the rate of transformation of aliphatic carbon double bonds (C = C) into single bonds (C–C) within the resin matrix and indirectly indicates the amount of residual monomer that may leach from the material [33]. The results revealed that the 0.5 wt% Chs NPs group was the only group to exhibit a statistically significant reduction in DC% relative to the control group (*p* < 0.001). This finding is consistent with Taher et al. [7], who reported a reduction in the DC% of 3D-printed clear aligner resin following incorporation of Chs NPs at concentrations up to 5 wt%. The reduced DC% observed at the higher Chs NPs concentration may be attributed to increased intermolecular interactions and crosslinking effects that restrict polymer chain mobility and interfere with the polymerization process [39]. In contrast, the 0.25 wt% Chs NPs group exhibited the highest mean DC% among all tested groups, although the increase was not statistically significant (*p* = 0.34). This finding may suggest that lower concentrations of Chs NPs can be incorporated without adversely affecting polymerization and may even facilitate polymer network formation without restricting chain mobility. Regarding the NZ-containing groups, DC% values remained comparable to those of the control group, indicating that NZ incorporation at the investigated concentrations did not adversely affect polymerization integrity. This finding partially contrasts with Holiel et al. [26], who reported a significant increase in DC% after incorporation of 0.25 wt% and 0.5 wt% NZ into 3D-printed hybrid ceramics. The discrepancy between the present findings and those reported by Holiel et al. [26] may be related to differences in resin composition, nanoparticle dispersion, printing parameters, or post-curing protocols, all of which can substantially influence polymerization kinetics and final conversion rates.

Following aging in artificial saliva, all study groups demonstrated a significant increase in DC%. This increase may be associated with artificial saliva adsorption and its plasticization effect, which may promote the leaching of residual monomers [25]. Nevertheless, DC% assessment alone does not substitute for direct biocompatibility or cytotoxicity testing. The present study did not include cytotoxicity evaluation, which should be recognized as an important limitation considering the intended intraoral application of these modified materials.

Clear aligners are susceptible to *S. mutans* biofilm accumulation, as *S. mutans* was selected as the primary bacterial model in this study due to its pivotal role as a pioneer colonizer and major etiological agent in dental plaque formation and subsequent white spot lesions [6]. In the present study, biofilm formation was evaluated using a crystal violet (CV) assay to quantify total adherent biofilm biomass. The results demonstrated a significant reduction in biofilm biomass in all modified groups compared with the control group (*p* < 0.001). However, the CV assay does not differentiate between bactericidal activity and anti-adhesive effects; therefore, additional investigations using live/dead bacterial assays and CFU analysis are required to better elucidate the underlying antimicrobial and anti-adhesive mechanisms of these modified materials. Notably, the 0.25 wt% NZ group exhibited the lowest OD after 48 h of *S. mutans* incubation, indicating reduced early-stage biofilm accumulation, which may be partially attributed to surface-mediated effects. SEM observations (Fig. 2c, d) revealed smoother and more uniform surface topography in the NZ-containing groups, which may have reduced initial bacterial adhesion, a critical step preceding biofilm maturation. This effect may be associated with more homogeneous nanoparticle dispersion within the resin matrix at lower NZ concentrations. In contrast, the 0.5 wt% NZ group exhibited the lowest OD and the lowest percentage increase in OD after 14 days of *S. mutans* incubation (Table 4). This finding may be attributed to the higher NZ concentration, which could provide a more sustained ion-release profile over extended incubation periods [40], in addition to the relatively smooth surface characteristics observed by SEM (Fig. 2e, f). To date, limited studies have investigated NZ incorporation into 3D-printed clear aligner resin. Nevertheless, the present findings are consistent with those of Velickovic et al. [41], who reported enhanced antimicrobial performance in NZ-modified mineral trioxide aggregate compared with unmodified material. The reduction in *S. mutans* biofilm biomass observed in the NZ-containing groups may be associated with multiple mechanisms, including reduced bacterial adhesion due to smoother surface topography, physical adsorption limiting microbial mobility, ion-exchange activity, and possible catalytic generation of reactive oxygen species (ROS) [42]. Regarding the Chs NPs-containing groups, the present findings are consistent with those reported by Taher et al. [7] and Alhuwaizi et al. [11], who demonstrated that incorporation of Chs NPs into 3D-printed clear aligner resin significantly reduced *S. mutans* viability. The observed reduction in biofilm biomass may be attributed to multiple mechanisms, including electrostatic interactions between positively charged Chs NPs and negatively charged teichoic acid of *S. mutans*. These interactions may compromise membrane integrity, increase permeability, and promote leakage of intracellular contents. Additional proposed mechanisms include interference with DNA replication, chelation of essential metal ions, and disruption of nutrient transport pathways [14]. In contrast to the NZ-containing groups, the Chs NPs groups demonstrated a concentration-dependent trend. The 0.5 wt% Chs NPs group exhibited lower OD values after 48 h of incubation compared with the 0.25 wt% Chs NPs group (Table 4), possibly due to enhanced concentration-dependent reduction in biofilm biomass [43]. SEM analysis also revealed clustered regions with increased polymer crosslinking in the 0.25 wt% Chs NPs group (Fig. 2g, h), which may have restricted nanoparticle release. However, this trend was reversed after 14 days of incubation. The higher Chs NPs concentration may have promoted greater release of residual components into the surrounding environment, corresponding with the lower DC% observed in this group (Table 1), which could theoretically influence long-term biofilm behaviour [44]. Regarding the hybrid group, the reduction in biofilm biomass was comparable to that observed in the 0.5 wt% Chs NPs group, with no statistically significant difference after either 48 h or 14 days of incubation (*p* = 0.39 and *p* = 0.10, respectively). Similarly, its performance after 48 h was comparable to that of the 0.25 wt% NZ group (*p* = 1.00). However, the hybrid formulation demonstrated lower biofilm reduction efficacy than the 0.5 wt% NZ group after 14 days of incubation.

Flexural strength represents a fundamental mechanical property influencing the clinical performance of clear aligners, as it significantly influences their ability to exert controlled orthodontic forces [45]. NZ-containing groups did not demonstrate a significant statistical difference in flexural strength relative to the control group (*p* = 0.34 and *p* = 0.10 for the 0.25 wt% NZ and 0.5 wt% NZ groups, respectively). This finding may be attributed to the relatively low NZ concentrations used and the adequate dispersion of nanoparticles within the polymer matrix. However, previous studies by Yaman et al. [46], Malic et al. [47], and Anwar et al. [48] reported that increasing NZ concentration may reduce flexural strength because of its intrinsic microporosity, hydrophilic nature, insufficient interfacial bonding, and non-uniform dispersion within the resin matrix. In contrast, the Chs NPs-containing groups demonstrated a significant increase in flexural strength compared with the control group (*p* < 0.001), consistent with Silva et al. [49]. This improvement may be related to enhanced interfacial bonding between Chs NPs and the carbonyl groups of the 3D-printed resin matrix, in addition to more favourable nanoparticle distribution and improved stress transfer within the polymer network [50]. The hybrid group exhibited the highest flexural strength among all tested groups, which may suggest a synergistic interaction between Chs NPs and NZ. This effect may be associated with improved NZ compatibility, enhanced interfacial adhesion, and more homogeneous nanoparticle distribution within the resin matrix through hydrogen-bond interactions facilitated by Chs NPs [51]. Following the aging protocol, all groups demonstrated a significant reduction in flexural strength, indicating that prolonged immersion in artificial saliva may induce hydrolytic degradation and plasticization of the polymer network, consistent with the findings reported by Mudhaffer et al. [27]. This deterioration may also be associated with increased water sorption and incomplete polymerization during the 3D-printing and post-curing processes, which facilitates interfacial weakening and subsequent failure at the resin–filler interface [52]. Although the 0.5 wt% NZ group exhibited the greatest reduction in flexural strength after aging in artificial saliva (Table 7), all recorded values remained above the minimum flexural strength requirement of 50 MPa specified by JIS T6528 and ISO 20795-2 for orthodontic polymer-based materials [53].

Surface hardness is another important mechanical property influencing the clinical performance of clear aligners, as it reflects resistance to surface indentation and is associated with wear resistance, surface roughness, colour stability, and plaque retention potential [54, 55]. The results demonstrated a significant enhancement in microhardness across all modified groups (*p* < 0.001) (Table 10). The microhardness increased in a concentration-dependent manner, with the 0.5 wt% modified groups exhibiting higher values than those with lower concentrations. This improvement may be attributed to the incorporation of nanoparticles, acting as reinforcing fillers that occupy interlayer gaps and microporosities and becoming integrated within the resin surface. In the NZ-containing groups, the increased microhardness is likely associated with the intrinsic hardness of NZ’s aluminosilicate crystalline structure and its presence on the surface, in agreement with the finding of Holiel et al. [26], who demonstrated that higher concentrations of NZ improve surface characteristics of 3D-printed hybrid ceramics, despite causing a slight reduction in bulk toughness. Similarly, Aljafery et al. [56] demonstrated that incorporating Ag-Zn zeolite into acrylic resin enhances its surface hardness. For the Chs NPs-containing groups, the improvement in microhardness may be attributed to enhanced interfacial adhesion between nanoparticles and the resin matrix, consistent with Tantavisut et al. [57], who reported increased hardness of polymethylmethacrylate bone cement following Chs NPs incorporation. The hybrid group exhibited the highest microhardness, suggesting a synergistic reinforcing effect between Chs NPs and NZ, resulting in improved structural reinforcement of the resin matrix [58]. In contrast to flexural strength results, all groups demonstrated a significant increase in microhardness following the aging protocol. This finding may be attributed to the interaction between the polymer network and artificial saliva, which can induce plasticization and increase chain mobility within the resin matrix. During Vickers indentation, this viscoelastic behaviour may facilitate greater elastic recovery upon load removal, resulting in smaller residual indentations and consequently higher apparent Vickers hardness values [59]. In addition, an increase in the degree of conversion and possible surface deposition of salivary mineral ions may further contribute to the observed increase in surface hardness [60].

Although this study provides valuable preliminary data, certain limitations must be considered. The in vitro nature of the design inherently lacks the dynamic complexity of the oral environment, particularly regarding salivary buffering, fluctuating pH levels, and functional masticatory stresses. Furthermore, the reliance on a mono-species S. mutans biofilm model serves as a simplified screening tool that does not capture the synergistic interactions of multi-species oral biofilms. Additionally, cytotoxicity, optical and aesthetic properties, such as transparency and color stability, were not assessed in this work. Since maintaining aesthetics is fundamental to clear aligner therapy, subsequent investigations are required to determine the impact of these nanoparticles on long-term optical performance and patient acceptance of the modified resin. To address these limitations, future research should focus on evaluating other mechanical and physical properties, including dimensional accuracy and cytotoxicity testing. Moreover, multi-species biofilm models with live/dead staining or CFU counts, as well as clinical trials, are necessary to confirm the safety and long-term performance of these modified aligners in the oral environment.

## Conclusions

Incorporation of nanozeolite (NZ) and chitosan nanoparticles (Chs NPs) into commercially available 3D-printed clear aligner resin (CR) resulted in a reduction in *Streptococcus mutans* biofilm biomass while maintaining or enhancing mechanical properties. Among the tested formulations, NZ-containing groups showed the most favorable reduction in biofilm biomass accumulation, whereas the hybrid group demonstrated the highest mechanical performance. Within the limitations of this in vitro study, these findings suggest that nanoparticle-modified resins may have potential applications in reducing biofilm formation and associated white spot lesions during clear aligner therapy.

## Data Availability

The data that support the findings of this study are available from the corresponding author upon reasonable request.

## Abbreviations

3D: Three-dimensional
Nanozeolite: NZ
Chitosan nanoparticles: Chs NPs
CR: Clear Resin
SEM: Scanning Electron Microscope
FTIR: Fourier-transform Infrared Spectroscopy
DC: Degree of Conversion
S. mutans: Streptococcus mutans
OD: Optical Density
FS: Flexural Strength
UTM: Universal Testing Machine
VHN: Vickers Hardness Number
Crystal violet: CV
STL: Standard Tessellation Language
CAD: Computer-aided Design
UTM: Universal Testing Machine
